# Secondary prevention in diabetic and nondiabetic coronary heart disease patients: Insights from the German subset of the hospital arm of the EUROASPIRE IV and V surveys

**DOI:** 10.1007/s00392-022-02093-0

**Published:** 2022-09-27

**Authors:** K. Ungethüm, S. Wiedmann, M. Wagner, R. Leyh, G. Ertl, S. Frantz, T. Geisler, W. Karmann, R. Prondzinsky, C. Herdeg, M. Noutsias, T. Ludwig, J. Käs, B. Klocke, J. Krapp, D. Wood, K. Kotseva, S. Störk, P. U. Heuschmann

**Affiliations:** 1 Institute of Clinical Epidemiology and Biometry, University of Würzburg, Josef-Schneider-Str. 2, 97080 Würzburg, Bavaria, Germany; 2 Charité - Universitätsmedizin Berlin, Corporate Member of Freie Universität Berlin and Humboldt-Universität Zu Berlin, Berlin, Berlin, Germany; 3 Department of Internal Medicine I, University Hospital Würzburg, Würzburg, Bavaria, Germany; 4 Kuratorium für Dialyse und Nierentransplantation E.V, Neu-Isenburg, Hesse, Germany; 5 Department of Clinical Research & Epidemiology, Comprehensive Heart Failure Center, University Hospital Würzburg, Würzburg, Bavaria, Germany; 6 Department of Internal Medicine III, University Hospital Halle, Martin-Luther-University Halle-Wittenberg, Saxony-Anhalt, Halle (Saale), Germany; 7 Department of Cardiology and Cardiovascular Disease, University Hospital Tübingen, Tübingen, Baden-Württemberg, Germany; 8 Department of Medicine, Klinik Kitzinger Land, Kitzingen, Bavaria, Germany; 9 Cardiology/Intensive Care Medicine, Carl Von Basedow Klinikum Merseburg, Merseburg, Saxony-Anhalt, Germany; 10 Medius Klinik Ostfildern-Ruit, Klinik für Innere Medizin, Herz- und Kreislauferkrankungen, Ostfildern-Ruit, Baden-Württemberg, Germany; 11 Department of Internal Medicine A, University Hospital Ruppin-Brandenburg (UKRB) of the Medical School of Brandenburg (MHB), Neuruppin, Brandenburg, Germany; 12 European Society of Cardiology, Sophia Antipolis, France; 13 Imperial College Healthcare NHS Trusts, London, UK; 14 National University of Ireland, Galway, Ireland; 15 Clinical Trial Center, University Hospital Würzburg, Würzburg, Bavaria, Germany

**Keywords:** Coronary heart disease, Diabetes Mellitus, Secondary Prevention, EUROASPIRE

## Abstract

**Background:**

Patients with coronary heart disease (CHD) with and without diabetes mellitus have an increased risk of recurrent events requiring multifactorial secondary prevention of cardiovascular risk factors. We compared prevalences of cardiovascular risk factors and its determinants including lifestyle, pharmacotherapy and diabetes mellitus among patients with chronic CHD examined within the fourth and fifth EUROASPIRE surveys (EA-IV, 2012–13; and EA-V, 2016–17) in Germany.

**Methods:**

The EA initiative iteratively conducts European-wide multicenter surveys investigating the quality of secondary prevention in chronic CHD patients aged 18 to 79 years. The data collection in Germany was performed during a comprehensive baseline visit at study centers in Würzburg (EA-IV, EA-V), Halle (EA-V), and Tübingen (EA-V).

**Results:**

384 EA-V participants (median age 69.0 years, 81.3% male) and 536 EA-IV participants (median age 68.7 years, 82.3% male) were examined. Comparing EA-IV and EA-V, no relevant differences in risk factor prevalence and lifestyle changes were observed with the exception of lower LDL cholesterol levels in EA-V. Prevalence of unrecognized diabetes was significantly lower in EA-V as compared to EA-IV (11.8% vs**.** 19.6%) while the proportion of prediabetes was similarly high in the remaining population (62.1% vs. 61.0%).

**Conclusion:**

Between 2012 and 2017, a modest decrease in LDL cholesterol levels was observed, while no differences in blood pressure control and body weight were apparent in chronic CHD patients in Germany. Although the prevalence of unrecognized diabetes decreased in the later study period, the proportion of normoglycemic patients was low. As pharmacotherapy appeared fairly well implemented, stronger efforts towards lifestyle interventions, mental health programs and cardiac rehabilitation might help to improve risk factor profiles in chronic CHD patients.

**Supplementary Information:**

The online version contains supplementary material available at 10.1007/s00392-022-02093-0.

## Introduction

Coronary heart disease (CHD) is the leading cause of death in Germany [1]. Modifiable risk factors contribute substantially to the development, recurrence, and outcome of cardiovascular diseases [2–4]. Particularly, lifestyle factors and comorbidities play an important role in primary and secondary CHD prevention [5], and appropriate control by lifestyle interventions might reduce recurrence, rehospitalisation, disability, and mortality in CHD patients [6, 7].

Targets of secondary CHD prevention are the promotion of weight loss in overweight and obese patients and control of blood pressure, lipid and glucose levels, by means of lifestyle and pharmaceutical treatment [5]. However, the importance of complimentary lifestyle interventions is often neglected in CHD patients [8–10]. As a consequence, comorbidities, such as hypertension, hyperlipidemia, or especially diabetes mellitus remain insufficiently controlled [8, 11]. In particular, diabetes mellitus represents a major risk factor for cardiovascular events, associated with unfavorable outcome including a negative impact on life expectancy [12, 13]. However, diabetes and its precursor states remain frequently undiagnosed in CHD patients [14–19]. Data on the dynamics of risk factor control in CHD patients in Germany are scarce. The European Action on Secondary and Primary Prevention by Intervention to Reduce Events (EUROASPIRE) survey program regularly evaluates medical treatment and risk factor control of chronic CHD patients in clinical practice in European countries. Within the German EUROASPIRE IV (EA-IV) subset, target levels of risk factor control including blood pressure, cholesterol levels, body weight and, in diabetic patients, HbA1c concentrations were frequently not achieved, although drug treatment appeared well implemented in the investigated population [11].

We compared prevalences of cardiovascular risk factors in chronic CHD patients based on the German subsets of the EA-IV (2012–13) and EA-V (2016–17) stud**ies** and analyzed determinants of prevalent overweight, hypertension, elevated LDL cholesterol and unrecognized dysglycemia. As diabetes mellitus is a major risk factor for recurring vascular events in CHD patients, analyses were also run stratified by known history in diabetes.

## Methods

### EUROASPIRE

The EUROASPIRE surveys are cross-sectional studies in patients with acute or chronic CHD, initiated by the EURObservational Research Programme (EORP) as part of the European Society of Cardiology. The EA-IV survey was carried out **in** 24 European countries with methodology and results published previously [10, 20–23]. The data of the EA-V survey were collected in 27 European countries, and methods and principal results of the hospital arm have been published previously [8].

### Study population

In brief, participants of EA-IV were enrolled in the geographical region of Würzburg representing one tertiary care hospital (University Hospital Würzburg) and one primary care hospital (Klinikum Kitzinger Land). In EA-V, for improving generalizability of the study results of the participating countries, data collection was expanded to additional geographical regions based on the size of the country. In Germany, patients were recruited in three geographical regions comprising one tertiary care hospital and one primary care hospital each: Halle/Saale (University Hospital Halle/Saale and Carl-von-Basedow Klinikum Merseburg), Tübingen (University Hospital Tübingen and medius Klinik Ostfildern-Ruit) and Würzburg (University Hospital Würzburg and Klinikum Kitzinger Land).

### Inclusion and exclusion criteria

Patients with chronic CHD, aged between 18 and 79 years at baseline, who had been hospitalized within 6 to 24 (for EA-V) or within 36 (for EA-IV) months prior to study inclusion, were invited to participate in the study. Reasons for admission (index event) were (i) elective or emergency coronary artery bypass grafting (CABG), (ii) elective or emergency percutaneous coronary intervention (PCI), (iii) acute myocardial infarction (MI) or (iv) acute myocardial ischemia (defined as acute coronary syndrome or angina pectoris). Eligible patients were identified through review of hospital records and were invited to participate by up to three postal letters. Prior study inclusion, all participants provided written informed consent.

### Data collection

Data collection was performed according to EUROASPIRE standards, defined by the comprehensive EA-IV and EA-V study protocols, standard operation procedures and training sessions provided to all participating study centers by EORP. In each region, one study center (University Hospital Halle, University Hospital Würzburg, University Hospital Tübingen) offered personal study visits. During the study visit, information on demographic characteristics, lifestyle factors, comorbidities, medical treatment and family history of diabetes and cardiovascular diseases were assessed during a standardized personal interview. Physical measurements included an oral glucose tolerance test (blood draw at 0 and 120 min), blood draw for determination of blood lipids and HbA1c, measurements of carbon monoxide in exhaled air, urine sampling to determine albumin/creatinine ratio and anthropometrics. Additional information on the index hospital stay, risk factors, medication prior the index event and diagnostics was collected by review of medical records. Detailed definitions for risk factors and comorbidities are given in the supplement. Prevalent diabetes mellitus was defined by self-report, intake of antidiabetic medication or documentation in medical records. According to the current guidelines by the German Diabetes Association, newly diagnosed diabetes at study visit was defined by fasting plasma glucose levels ≥ 126 mg/dl (7.0 mmol/l), or a 2-h post-load plasma glucose levels ≥ 200 mg/dl (11.1 mmol/l) during oral glucose tolerance test, or an occasional plasma glucose levels ≥ 200 mg/dl (11.1 mmol/l). A prediabetic state was defined by impaired fasting glucose (fasting glucose 100 to 125 mg/dl (5.6–6.9 mmol/l)) (IGT) and/or impaired glucose tolerance (IGT and 2-h post-load plasma glucose 140–199 mg/dl (7.8–11.0 mmol/l) during the study visit. Patients with glucose levels within the physiological/prediabetic spectrum and HbA1c levels ranging from 6.5 to 7% were not considered as diabetic [24].

### Data management

All data contained in the EUROASPIRE IV and V study protocol were collected using electronic case report forms by subject-specific unique identifiers. The central database was located at the data management center (EURObservational Research Program (EORP), ESC, Sophia-Antipolis, France), where data was initially reviewed for completeness and plausibility, before a copy of the German dataset was provided for the analyses presented.

### Data analysis

To describe the study population and analyse differences between different subgroups, χ^2^ test, Fisher’s exact test, Mann–Whitney U test and Kruskal–Wallis test were applied as appropriate. To assess determinants of risk factor control and unrecognized diabetes mellitus, the EA-IV and EA-V study populations were pooled for increasing power of the models, and logistic regression models were calculated. The probability of having diabetes in the EA-IV and EA-V study population was estimated by logistic regression including sex, age and the type of the index event, and probabilities were estimated strata-wise [25]. To analyze factors associated to reach the target levels for BMI, blood pressure and LDL cholesterol, a previously published model by Cooney et al. [26] was adapted and modified based on univariate analyses in the present study population and clinical knowledge. The final model included age, sex, institution (tertiary vs. primary care), education, symptoms of depression, symptoms of anxiety, previous diagnosis of CHD prior to the index event, type of the index event, completion of cardiac rehabilitation, professional support (cardiologist, general practitioner, physician), and study affiliation. For determining potential predictors of unrecognized diabetes, a model of Rathmann et al. [27] was used as basic model and also modified based on analyses in the present population and clinical knowledge. The final model included age, sex, BMI, LDL cholesterol, lipid-lowering medication, family history in diabetes, hypertension, ever smoking, months between the index event and interview, regular vigorous physical activity, change of diet, type of the index event and study affiliation.

Determinants are given as Odds Ratio (OR) with 95% confidence intervals (CI). Statistical significance was set at α = 0.05 (two-tailed). Statistical analysis was performed using SAS 9.4 (SAS Institute Inc., Cary, NC, USA) and R 3.5.1 (RFoundation for Statistical Computing, Vienna, Austria).

### Sensitivity analyses

As the study participants were recruited in different regions in EA-V, while in EA-IV patients were exclusively recruited in the region of Würzburg, we repeated the comparison of prevalences including patients from the Würzburg region, only (*n* = 215).

### Sample size considerations

Sample size calculations were based on the assumption to allow the estimation of country-specific prevalences with at least 5% precision and 95% confidence level. To achieve this precision, it was aimed to recruit at least 400 patients in each study, in EAV (200 in the region of Würzburg, and 100 in the regions of Halle and Tübingen, each) and EA-IV (400 in the region of Würzburg) in Germany.

### Ethical approval

EA-IV and EA-V were approved by the Ethics Committee of the Medical Faculty of the University of Würzburg (vote 58/12) and the ethics comittees of the corresponding centers (Halle vote 2016–72; Tübingen vote 385/2016BO2). The data protection officers of the University Würzburg and the University Hospital Würzburg approved the data protection concept. The international study was registered at Clinicaltrials.gov (NCT03511885).

## Results

### Study population

**Fig. 1:**
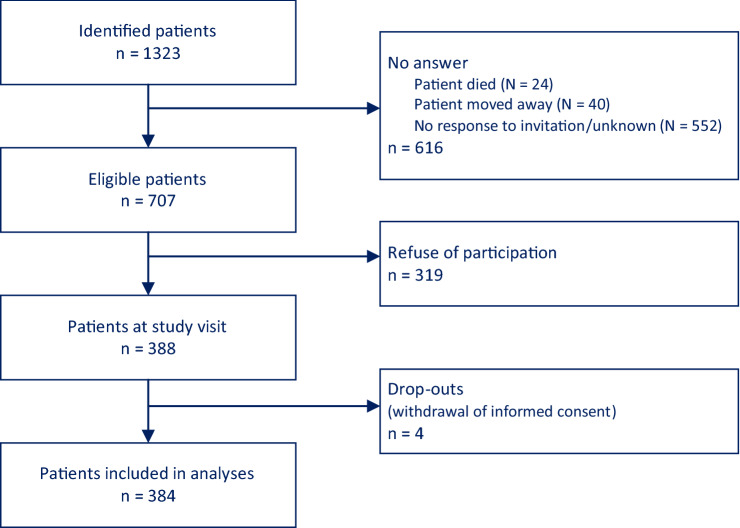
Flowchart of the EUROASPIRE V study population

From 08/2012–03/2013, 536 EA-IV study participants from Würzburg (*n* = 498) and Kitzingen (*n* = 38), median age 68.7 (quartiles 61.7, 74.3) years, 82.3% men, were interviewed and examined [11]. In EA-V, out of 1323 invited patients, 384 patients were included between 10/2016–06/2017: from Halle (*n* = 75), Merseburg (*n* = 25), Tübingen (*n* = 60), Ostfildern-Ruit (*n* = 9), Würzburg (*n* = 202), and Kitzingen (*n* = 13). Median age was 69.0 years (quartiles 63.9, 75.8), and 81.3% were men (Fig. 1). The study populations are characterized and compared in Table 1.

**Table 1:** Characteristics of participants in German EA-V and EA-IV surveys by history of diabetes

	EA-IV				EA-V		EA-IV vs. EA-V
	All patients (*n* = 536)^a^	No history of diabetes (*n* = 369)	History of diabetes (*n* = 167)	All patients (*n* = 384)	No history of diabetes (*n* = 273)	History of diabetes (*n* = 107)	*P*
Age, median (IQR)	68.7 (61.7–74.3)	68.4 (61.7–73.8)	69.7(61.7–75.6)	69.0 (63.9–75.8)	68.8 (62.9–75.5)	70.3 (64.9–76.2)	**0.03**
Age categories, *n* (%)							**0.003**
< 65.0 years	192 (35.8)	139 (37.7)	53 (31.7)	109 (28.7)	81 (29.7)	28 (26.2)	
65.0–69.9 years	109 (20.3)	73 (19.8)	36 (21.6)	95 (25.0)	71 (26.0)	24 (22.4)	
70.0–74.9 years	115 (21.5)	84 (22.8)	31 (18.6)	63 (16.6)	46 (16.9)	17 (15.9)	
≥ 75 years	120 (22.4)	73 (19.8)	47 (28.1)	113 (29.7)	75 (27.5)	38 (35.5)	
Sex, male, *n* (%)	441 (82.3)	301 (81.6)	140 (83.8)	309 (81.3)	216 (79.1)	93 (86.9)	0.77
Education, *n* (%)							** < 0.0001**
Primary school or less	9 (1.7)	6 (1.6)	3 (1.8)	3 (0.8)	2 (0.7)	1 (0.9)	
Secondary school or high school	32 (6.0)	22 (6.0)	10 (6.0)	137 (36.2)	100 (36.8)	37 (34.9)	
Vocational or technical training	395 (73.7)	271 (73.4)	124 (74.3)	146 (38.6)	104 (38.2)	42 (39.6)	
University or postgraduate	100 (18.7)	70 (19.0)	30 (18.0)	92 (24.3)	66 (24.3)	26 (24.5)	
Reason for admission, *n* (%)							** < 0.0001**
Elective CABG	83 (15.5)	53 (14.4)	30 (18.0)	75 (19.5)	46 (16.9)	28 (26.2)	
Elective PCI or stent	370 (69.0)	261 (70.7)	109 (65.3)	121 (31.5)	86 (31.5)	32 (29.9)	
Acute MI	28 (5.2)	19 (5.2)	9 (5.4)	142 (36.9)	108 (39.6)	34 (31.8)	
Acute myocardial ischemia or unstable angina	55 (10.3)	36 (9.8)	19 (11.4)	46 (12.0)	33 (12.1)	13 (12.2)	
Years between index event and study visit, median (IQR)	1.8 (1.1–2.5)	1.8 (1.1–2.5)	1.8 (1.1–2.5)	1.2 (0.9–1.5)	1.2 (0.8–1.6)	1.0 (0.9–1.4)	** < 0.0001**
Completion of a cardiac rehabilitation program, *n* (%)	263 (49.2)	181 (49.1)	82 (49.4)	190 (49.6)	135 (49.5)	54 (50.5)	0.89
Smoking, *n* (%)							
Never smoking	182 (34.0)	138 (37.4)	44 (26.4)	113 (29.4)	91 (33.3)	22 (20.6)	0.15
Current smoking	65 (12.1)	47 (12.7)	18 (10.8)	60 (15.6)	46 (16.9)	12 (11.2)	0.13
Regular physical activity, *n* (%)^**b**^	46 (13.2)	43 (17.6)	3 (2.9)	48 (21.2)	35 (20.0)	11 (22.0)	**0.01**
Change of diet within the previous 6 months, *n* (%)	530 (98.9)	364 (98.6)	166 (99.4)	341 (88.8)	239 (87.6)	100 (93.5)	** < 0.0001**
Heart failure, *n* (%)	79 (14.7)	45 (12.2)	34 (20.4)	74 (19.4)	48 (17.7)	25 (23.4)	0.06
Coronary heart disease prior the index event, *n* (%)	353 (65.9)	241 (65.3)	112 (67.1)	140 (36.7)	85 (31.4)	54 (50.5)	** < 0.0001**
Duration of CHD, years, median (IQR)	2.9 (1.9–9.0)	2.7 (1.9–5.6)	3.8 (2.1–14.4)	2.1 (1.4–7.5)	2.0 (1.4–3.8)	3.2 (1.5–13.1)	** < 0.0001**
Peripheral artery disease, *n* (%)	36 (6.7)	19 (5.2)	17 (10.2)	17 (10.9)	10 (10.5)	7 (11.7)	0.08
History of stroke/TIA, *n* (%)	46 (8.6)	25 (6.8)	21 (12.6)	24 (6.3)	14 (5.2)	10 (9.4)	0.20
Symptoms of depression (HADS), *n* (%)	97 (18.1)	61 (16.5)	36 (21.6)	58 (15.1)	35 (12.8)	23 (21.5)	0.23
Symptoms of anxiety (HADS), *n* (%)	148 (27.9)	102 (27.8)	46 (28.1)	82 (21.9)	59 (21.9)	23 (22.8)	0.04

^a^Partly published by Wagner et al. [11]; ^b^In patients without long-standing illness, disability or infirmity (EA-V: *n* = 215; EA-IV: *n* = 330); *PCI* Percutaneous coronary intervention, *CABG* Coronary artery bypass graft, *MI* Myocardial infarction, *CHD* Coronary heart disease, *TIA* Transient ischemic attack, *HADS* Hospital Anxiety and Depression Scale

p-values marked in bold are statistically significant at a two-tailed α-level of 0.05

In EA-V, patients were older and the predominant reasons for admission were acute MI and elective PCI, while in EA-IV, the index event was most frequently an elective PCI. Determined by respective inclusion criteria, time between index event and study visit was shorter in EA-V than in EA-IV. Further, EA-V patients were more frequently physically active, and less likely to have tried changing diet during the 6 months prior baseline investigation. EA-V participants had a lower proportion of manifested CHD prior the index event and, in this case, a shorter duration of CHD. Symptoms of anxiety were more frequently prevalent in EA-IV than in EA-V. In EA-IV and EA-V, 167 (31.1%) and 107 (27.9%) patients had a history of diabetes, respectively.

### Differences in risk factor control between EA-IV and EA-V

The use of medication and measures of risk factor control are given in Table 2. The use of single aspirin was lower in EA-V as compared to EA-IV, but the use of aspirin combined with clopidogrel or another platelet inhibitor was higher. The proportion of anticoagulation was higher, while the use of cardioprotective/antihypertensive medication and lipid-lowering drugs was lower in EA-V. Regarding risk factor control, overweight/obesity, elevated blood pressure, and elevated LDL cholesterol had a high prevalence in EA-V, but no statistically significant differences between the entire study populations were observed for BMI and elevated blood pressure at baseline. However, median LDL cholesterol levels were substantially lower in EA-V and target levels of LDL cholesterol < 2.6 mmol/l and < 1.8 mmol/l were more frequently reached. In line, target levels of LDL cholesterol were more frequently reached under lipid-lowering therapy.

**Table 2:** Risk factor control in diabetic and nondiabetic CHD patients

	All patients				No history of diabetes			History of diabetes		
	EA-IV (*n* = 536)^a^		EA-V (*n* = 384)	*P*	EA-IV (*n* = 369)	EA-V (*n* = 273)	*P*	EA-IV (*n* = 167)	EA-V (*n* = 107)	*P*
*Medication*										
No medication, *n* (%)	4 (0.8)		3 (0.8)	>0.99	4 (1.1)	3 (1.1)	>0.99	0 (0.0)	0 (0.0)	NA
Platelet inhibitors, *n* (%)	477 (89.0)		331 (86.7)	0.28	333 (90.2)	238 (87.2)	0.22	144 (86.2)	90 (84.9)	0.76
Aspirin only	362 (67.5)		223 (58.4)	**0.004**	258 (69.9)	163 (59.7)	**0.007**	104 (62.3)	57 (53.8)	0.16
Clopidogrel	13 (2.4)		8 (2.1)	0.74	9 (2.4)	5 (1.8)	0.60	4 (2.4)	3 (2.8)	>0.99
Combined antiplatelet regimen	98 (18.3)		100 (26.2)	**0.004**	64 (17.3)	70 (25.6)	**0.01**	34 (20.4)	30 (28.3)	0.13
Anticoagulation, *n* (%)	26 (4.9)		54 (14.3)	**<0.0001**	14 (3.8)	36 (13.3)	**<0.0001**	12 (7.2)	17 (16.4)	**0.02**
Antihypertensive medication, *n* (%)	513 (99.2)		361 (95.3)	**0.0001**	350 (98.9)	256 (94.1)	**0.0008**	63 (100.0)	102 (98.1)	0.15
Betablocker	447 (86.5)		300 (79.2)	**0.004**	305 (86.2)	210 (77.2)	**0.004**	142 (87.1)	87 (83.7)	0.43
ACE inhibitor	289 (55.9)		170 (45.1)	**0.001**	188 (53.1)	123 (45.4)	0.06	101 (62.0)	46 (44.2)	**0.005**
Angiotensin II receptor blocker	153 (29.6)		144 (38.0)	**0.008**	106 (29.9)	100 (36.8)	0.07	47 (28.8)	42 (40.4)	**0.05**
Calcium channel blocker	121 (23.4)		96 (25.4)	0.49	66 (18.6)	60 (22.1)	0.29	55 (33.7)	35 (33.7)	0.99
Other^**b**^	57 (11.0)		37 (9.8)	0.54	33 (9.3)	20 (7.4)	0.38	24 (14.7)	17 (16.4)	0.72
Diuretic, *n* (%)	242 (45.2)		181 (47.5)	0.50	140 (37.9)	112 (41.0)	0.43	102 (61.5)	69 (65.1)	0.54
Lipid-lowering drug, *n* (%)	454 (99.1)		337 (88.2)	**<0.0001**	311 (98.7)	234 (85.7)	**<0.0001**	143 (100.0)	100 (94.3)	**0.006**
Statin	446 (97.4)		333 (87.2)	**<0.0001**	308 (87.8)	232 (85.0)	**<0.0001**	138 (96.5)	98 (92.5)	0.15
Glucose-lowering drugs, *n* (%)								131 (78.4)	93 (87.7)	0.05
*Risk factors*										
Body weight										
Body mass index (kg/m^2^), median (IQR)					27.8 (25.8–30.4)	28.4 (25.7–31.2)	0.20	30.3 (27.5–33.5)	29.8 (26.8–32.7)	0.30
Body weight category, *n* (%)							**0.04**			0.24
Normal weight					64 (17.6)	56 (20.7)		12 (7.2)	14 (13.3)	
Overweight					195 (53.6)	118 (43.5)		64 (38.6)	40 (38.1)	
Obesity					105 (28.9)	97 (35.8)		90 (54.2)	51 (48.6)	
Blood pressure										
Hypertension at baseline, *n* (%)	232 (43.4)		158 (41.4)	0.55	150 (40.8)	106 (38.8)	0.62	82 (49.1)	50 (47.6)	0.81
Pts on antihypertensive medication	219 (42.8)		146 (40.7)	0.54	139 (39.8)	98 (38.3)	0.70	80 (49.1)	47 (47.0)	0.74
*LDL cholesterol*										
LDL-C (mmol/l), median (IQR)	2.5 (2.1–3.1)		2.2 (1.8–2.7)	**<0.0001**	2.6 (2.2–3.2)	2.2 (1.8–2.8)	**<0.0001**	2.4 (1.9–2.9)	2.1 (1.7–2.4)	**0.001**
LDL-C ≥2.6 mmol/l (100 mg/dl), *n* (%)	230 (45.5)		104 (29.3)	**<0.0001**	180 (50.3)	87 (33.6)	**<0.0001**	50 (34.0)	16 (17.4)	**0.005**
Pts on lipid-lowering therapy	173 (40.4)		70 (22.4)	**<0.0001**	131 (43.4)	56 (25.2)	**<0.0001**	42 (33.3)	13 (14.9)	**0.003**
LDL-C ≥1.8 mmol/l (70 mg/dl), *n* (%)	451 (89.3)		263 (74.1)	**<0.0001**	330 (92.2)	198 (76.5)	**<0.0001**	121 (82.3)	61 (66.3)	**0.005**
Pts on lipid-lowering therapy	376 (87.9)		221 (70.8)	**<0.0001**	276 (91.4)	161 (72.5)	**<0.0001**	100 (79.4)	57 (65.5)	**0.02**
HbA1c										
HbA1c (%), median (IQR)					5.6 (5.4–5.8)	5.5 (5.3–5.7)	<0.0001	6.6 (6.1–7.6)	6.7 (6.3–7.3)	0.42
HbA1c ≥6.5%, *n* (%)					10 (2.7)	3 (1.2)	0.17			
HbA1c ≥7.0%, *n* (%)								64 (39.0)	42 (42.9)	0.54
Newly diagnosed diabetes mellitus, *n* (%)^**c**^					72 (19.6)	31 (11.8)	0.01			
Fasting glucose ≥7.0 mmol/l (126 mg/dl)					59 (16.4)	27 (10.7)	0.04			
2-h glucose during OGTT ≥11.1 mmol/l (200 mg/dl)					17 (5.7)	9 (3.7)	0.29			
Prediabetic state/Gray zone, *n* (%)^**c**^					225 (61.0)	172 (62.1)	0.77			
Impaired fasting glucose (IFG)					206 (57.2)	164 (64.8)	0.31			
Impaired glucose tolerance (IGT)					42 (11.4)	50 (18.1)	0.02			
5.7% ≤HbA1c <6.5%					164 (44.8)	78 (29.32)	<0.0001			
6.5% ≤HbA1c <7.0% and fasting glucose <7.0 mmol/l and 2-h glucose during OGTT <11.1 mmol/l					2 (0.6)	2 (0.8)	0.76			

^a^Partly published by Wagner et al. [11]; ^b^Nitrates, I_*f*_ channel blocker, ranolazine, renin inhibitor;^c^ Patients without information on history in diabetes (*n* = 4) are included in these analyses. *ACE* Angiotensin-converting enzyme *LDL-C* Low-density lipoprotein cholesterol *OGTT* Oral glucose tolerance test

p-values marked in bold are statistically significant at a two-tailed α-level of 0.05

Differences in use of platelet inhibitors, overall use of antihypertensive medication, and statins as well as BMI were only observed in patients without known history in diabetes but not in diabetic patients.

**Fig. 2:**
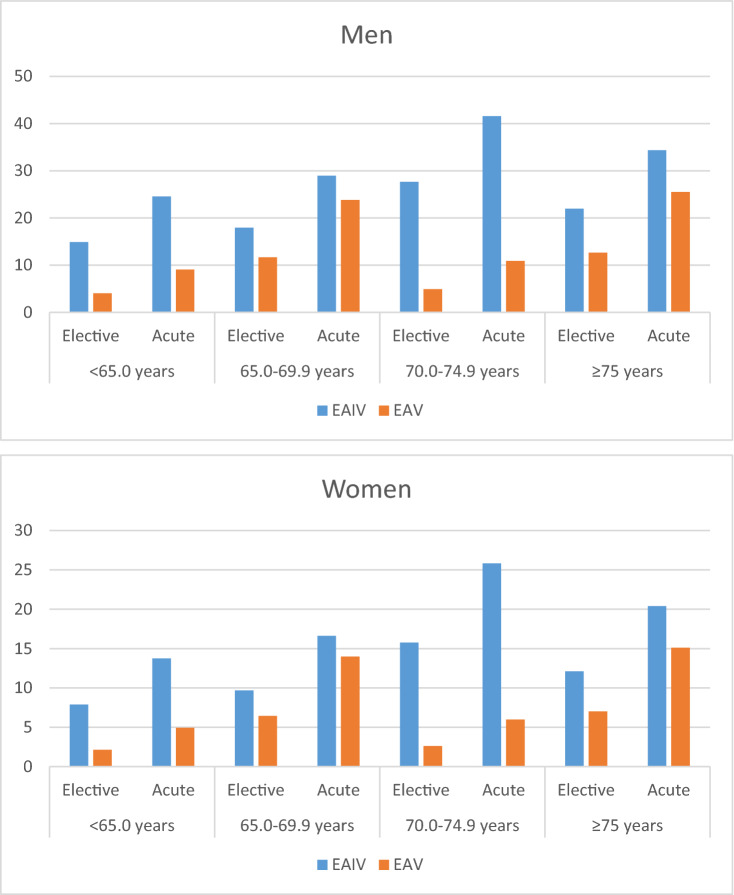
Probabilities of having unrecognized diabetes mellitus in EA-IV and EA-V, stratified by age, type of the index event, and gender

HbA1c levels were slightly, but statistically significantly lower in EA-V in patients without a history of diabetes. The proportion of patients without a positive history, but with evidence for existing diabetes based on glucose measurements during an oral glucose tolerance test, was significantly lower in the EA-V study population compared to EA-IV. Of them, 52.8% in EA-IV and 48.4% in EA-V were older than 70 years. **In EA-V,** in total, 62.1% of nondiabetics were prediabetic. In EA-IV, the proportion of prediabetic patients was 61.0%. The probability of unrecognized diabetes in different subgroups, stratified by age, gender and type of the index event, was substantially lower in EA-V across all strata (Fig. 2, Supplemental Table S2). Men and patients with an acute index event had a higher probability of suffering from diabetes than women and patients with an elective index event.

For sensitivity analyses, only patients from the Würzburg region were compared. There were no statistically significant differences in physical activity and symptoms of anxiety, but the proportion of heart failure was statistically significantly higher in EA**-**V (data not shown).

### Determinants of risk factor control

**Fig. 3:**
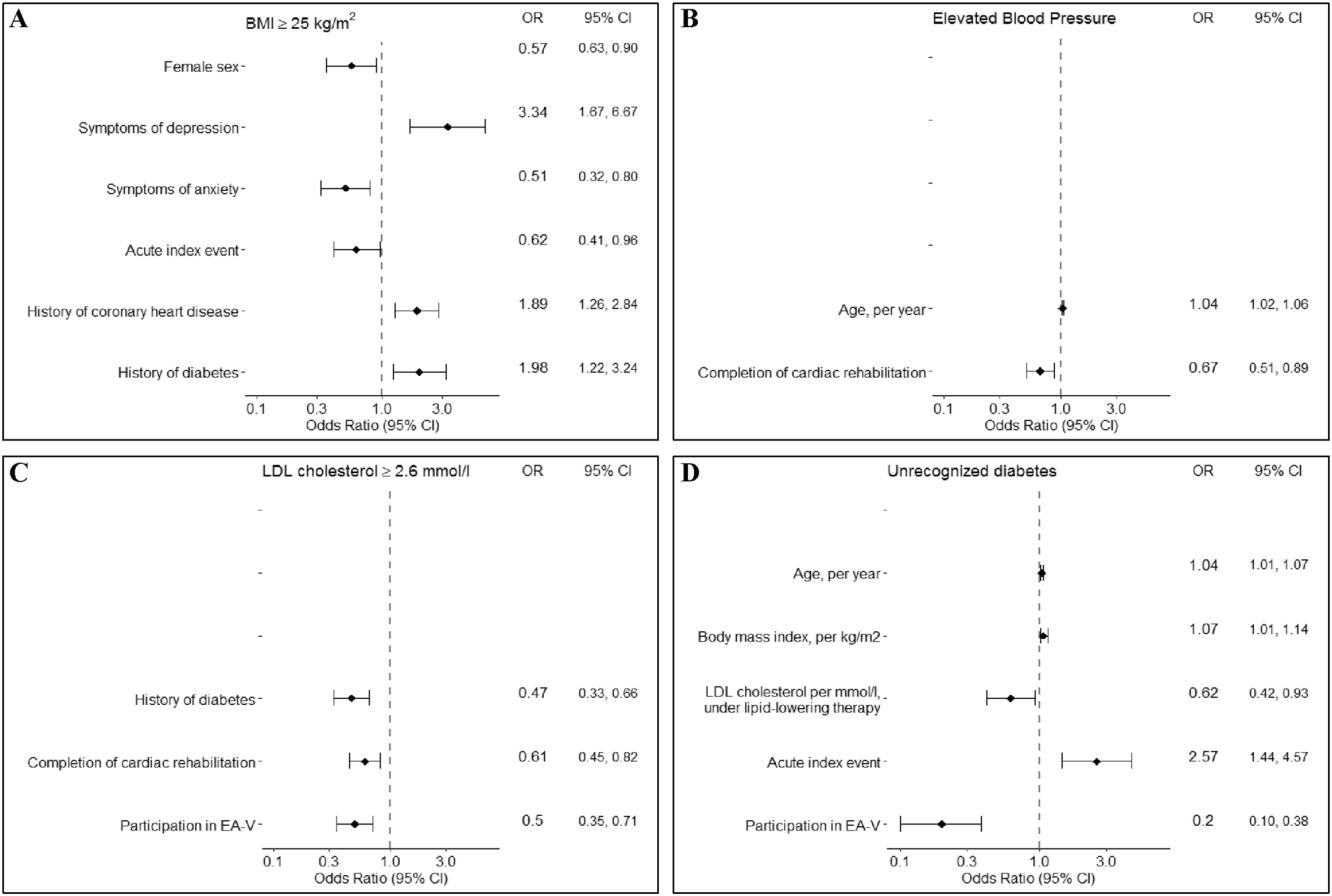
Determinants of prevalent risk factors at baseline; pooled analysis of EA-IV and EA-V participants. **A**, **B**, **C**: Model adjusted for age, sex, institution (tertiary vs. primary care), education, depression, anxiety previous diagnosis of coronary heart disease prior to the index event, type of index event (elective/acute), completion of cardiac rehabilitation, professional support (cardiologist, general practitioner, physician), study affiliation. Modified model, primarily published by Cooney et al. [26]. **D**: Model adjusted for age, sex, body mass index, LDL cholesterol, lipid-lowering medication, family history in diabetes, hypertension, ever smoking, months between the index event and interview, regular vigorous physical activity, change of diet, study affiliation. Model based on the KORA basic model for prediction of diabetes. [27]

Determinants for the presence of risk factors were analyzed over the whole study population (EA-IV and EA-V). The results are shown in Fig. 3A–C. Overweight/obesity were positively associated with symptoms of depression, history of CHD and history of diabetes mellitus, but inversely associated with female sex, an acute index event and symptoms of anxiety. Hypertension at the time of the interview was positively associated with higher age and inversely associated with completion of a cardiac rehabilitation program. LDL cholesterol levels ≥ 2.6 mmol/l were inversely associated with history of diabetes mellitus, completion of a cardiac rehabilitation program, and EA-V study affiliation.

In pooled analyses of both study samples, determinants of unrecognized diabetes were increasing age, higher BMI, decreasing LDL cholesterol under lipid-lowering therapy, an acute index event, and EA-V study affiliation (for details refer to Fig. 3D).

## Discussion

In the EA-V study population, a majority of patients received pharmaceutical treatment according to current guidelines in terms of platelet inhibition, cardioprotective and antihypertensive medication, and lipid-lowering drugs, especially statins. However, treatment targets for body weight, LDL cholesterol and blood pressure were frequently not met, and measures of lifestyle changes were insufficiently implemented. Compared to the earlier EA-IV study, a positive trend was observed regarding regular vigorous physical activity, while a negative trend was shown for the attempt to change diet. Compared with the EA-IV study population, improvement in reaching treatment targets for LDL cholesterol and HbA1c, but no differences in weight and blood pressure control were found. The use of cardioprotective and antihypertensive medication was lower in EA-V although, the index event was more often an acute event in this study population. Differences in risk factor prevalence did not differ materially between diabetic and nondiabetic patients.

Both studies followed standardized study protocols provided by the international study coordination. The protocols differed only slightly between the study periods despite a longer time span between index event and baseline investigation in EA-IV and recruitment in three different regions in EA-V. The variables investigated in the present study were collected and defined identically. Comparability of the study populations might be further limited due to differing baseline characteristics, including higher age, a significant higher prevalence of an acute index event, and shorter duration of CHD in EA-V patients.

The results of the German EA-V subset are in line with the findings of the entire EA-V study population (*n* = 8261) with participants from 27 countries [8]. In the international study population, overweight and obesity had a prevalence of 82% (Germany: 81.6%), 42% had elevated blood pressure (Germany: 41.4%) and 37% had LDL cholesterol levels ≥ 2.5 mmol/l (Germany: 29.3%) [8]. Diabetes was newly diagnosed at study visit in 12.5% (Germany: 11.8%) [17].

One of the main reasons for not reaching the target levels might be the older age of the patients. The 2016 ESC guidelines on cardiovascular prevention suggest that the target levels for management of diabetes, hypertension and hyperlipidemia in elderly persons (aged 65 years and older) should be addressed, but eventually modified and relaxed individually [5]. Lifestyle changes might be more difficult to implement in these patients due to multimorbidity/frailty, especially in case of increasing physical activity to reduce body weight, improve lipid and blood pressure levels and mortality risk [5, 28]. As hypertension, diabetes mellitus and dyslipidemia are the main risk factors for recurrent events and cardiovascular mortality risk [29], their management in terms of pharmaceutical treatment as well as appropriate lifestyle changes is crucial for secondary prevention and needs to be stressed – also in elderly chronic CHD patients.

In the German study population, women were less likely to be overweight or obese compared to men. This is not in line with the findings from international EA populations, where the proportion of obesity was constantly higher in women compared to men [8, 10]. However, the proportion of women was lower in the German study population (19%) compared to the international EA V population (26%), which might limit the comparability.

In contrast to findings of the EA-III survey [26], symptoms of anxiety and depression were associated with overweight/obesity in chronic CHD patients in the studied population. The association of depression with an increased risk for overweight/obesity was also described by a previous meta-analysis of observational studies [30]. In contrast, the association of anxiety and overweight/obesity was inverse in the studied population. A recent meta-analysis found a positive association of anxiety and overweight/obesity, however, this was not specific for an elderly and chronically diseased population [31]. Both, depression and anxiety are established risk factors for cardiovascular mortality, thus, screening and interventions are recommended for primary and secondary prevention of cardiovascular events [5, 32]. As manifested risk factors, elevated BMI levels were positively associated with history in CHD and diabetes mellitus in our study population. Hypertension was associated with increasing age as expected [33]. Interestingly, the completion of a cardiac rehabilitation program was associated with both, reaching a LDL cholesterol level lower than 2.6 mmol/l and blood pressure levels below the diagnostic level of hypertension. The type of cardiac rehabilitation was not specified in the questionnaire, thus, any cardiac rehabilitation program for CHD might have beneficial effects [34]. Lower LDL cholesterol levels were moreover associated with history in diabetes mellitus and EA-V study affiliation. This association might partly be explained by the significantly higher use of statins within diabetic EA-V participants.

Dysglycemia, including diabetes but also its preceding states, are associated with an increased risk for micro- and macrovascular events, requiring a multifactorial approach to reach the, partly more stringent, target levels of weight, blood pressure and lipids for cardiovascular prevention [5, 17, 35]. The prevalence of unrecognized diabetes decreased significantly between 2012–13 and 2016–17. In comparison with the international study population, the proportion of patients with undiagnosed diabetes was slightly lower in the German study population [17]. This trend might be caused by increasing awareness of the importance to control diabetes in CHD patients [36]. Undiagnosed diabetes was associated with increasing age, higher BMI, and an acute MI index event. Moreover, and decreasing lipid levels under use of lipid-lowering drugs. Statin use is known to be time- and dose-dependently associated with incident diabetes [37, 38]. These findings emphasize the necessity of regular glucose monitoring and higher awareness for a healthy lifestyle in chronic CHD patients, especially in the prediabetic state. Only a minority of the investigated populations had normal glucose metabolism.

The presented data lend support to further enhanced efforts in health education in Germany, in particular for patients with CHD, but also for primary prevention. Especially, support for the adoption of a healthier lifestyle, respecting factors like age, multimorbidity, disability, but also socioeconomic factors, might help to improve the situation. The use of structured cardiac rehabilitation after hospital discharge should be better implemented as it is suited to improve risk factor control. Currently, only half of the patients completed such a program.

## Strengths and limitations

The major strength of this study is the standardization of data collection in the investigated study populations over time due to standardized study protocols, standardized operation protocols and trainings for the examining study personal. The generalizability of the study results for Germany increased in EA-V compared to previous EA projects, because six sites from three different geographical areas were jointly investigated allowing to sample CHD patients with a heterogeneous risk profile, yet fairly representative for Germany. Nevertheless, the major limitations of this study include the possibility of selection bias, as patients more aware of their health status might have been more inclined to participate after postal invitation, and the response rate was relatively low. As a consequence, the prevalences of risk factors might have been underestimated [8]. In EA-V, only 384 patients were included in the analyses (vs. 536 in EA-IV). Secondly, comparability between the studies (EA-IV and EA-V) is limited due to different inclusion criteria regarding the time between the index event and study examinations. A previous study with similar inclusion criteria as the EA studies, has demonstrated that outpatients after an acute event are more likely to reach risk factor targets compared to patients with history of an elective CABG or PCI [39]. However, as guidelines on secondary prevention recommend similar risk factor targets for all chronic CHD patients, and secondary prevention is a continuous process not limited by time, comparison of the presented data might be justified. Thirdly, the analyses were cross-sectional, giving only one time point between 6 and 24 or 36 months, respectively, after the index event. To obtain a more general picture of the quality of secondary prevention in Germany, a longitudinal study design, starting with hospital discharge would be desirable.

## Conclusion

Compared with the EA-IV study population, LDL cholesterol levels were lower in EA-V. This indicates an important step towards sufficient secondary prevention in CHD. The use of pharmacotherapy was high. Nevertheless, targets of body weight, glucose levels, blood pressure, and comorbidities were insufficiently controlled over the combined observation period. As pharmacotherapy is well implemented, improvements in lifestyle interventions including increased regular physical activity, successful change of diet and smoking cessation but also mental health interventions and cardiac rehabilitation programs might aid to further improve the cardiovascular risk profile in chronic CHD patients. Although dysglycemia increases the risk for cardiovascular re-events and mortality, the prevalence of normoglycemia was low. The data of the EA-IV and EA-V study populations demonstrate the urge for improvements in continuous glucose monitoring and multifactorial CVD prevention.

## Supplementary Information

Below is the link to the electronic supplementary material.

Supplementary file1 (DOCX 27 KB)
