# Supplementary material for: Secondary prevention in diabetic and nondiabetic coronary heart disease patients: Insights from the German subset of the hospital arm of the EUROASPIRE IV and V surveys
Source: Clin Res Cardiol. 2022 Sep 27;112(2):285–98. doi: 10.1007/s00392-022-02093-0 (PMC9898414; doi:10.1007/s00392-022-02093-0)
Supplement: Supplementary file 1 — Supplementary file1 (DOCX 27 KB) [file 392_2022_2093_MOESM1_ESM.docx]

**Table S1 – Variable definitions**

| **Variable** | **Definition** |
| --- | --- |
| Known diabetes mellitus | *At least one of the following* :  Recorded history of diabetes  Anti-diabetic medication  Self reported at interview |
| Newly diagnosed diabetes mellitus | *At least one of the following* :  Fasting blood glucose ≥126 mg/dl (≥7.0 mmol/ [A1]  2-hours plasma glucose ≥200 mg/dl (≥11.1 mmol/l) [A1] |
| Prediabetes | *At least one of the following* :  Impaired fasting glucose (fasting blood glucose 100-125 mg/dl (5.6-6.9 mmol/l)) [A1]  Impaired glucose tolerance (fasting blood glucose <125 mg/dl (5.6 mmol/l) and 2-hours plasma glucose 140-199 mg/dl (7.8-11.0 mmol/l)) [A1]  HbA1c 5.7-6.4% (39-47 mmol/mol Hb) [A1] |
| Dyslipidaemia | LDL ≥1.8 mmol/L (≥ 70 mg/dl) [[A2](#_ENREF_5)] |
| Hypertension [A3] | ≥140/90 mmHg  *Patients with diabetes*  should be ≥140/85 mmHg |
| Heart failure | *At least one of the following*  recorded history of HF  Self reported at interview |
| Symptoms of anxiety | Hospital Anxiety and Depression Score [A4], Anxiety Subscale ≥8 |
| Symptoms of depression | Hospital Anxiety and Depression Score[A4], Depression Subscale ≥8 |
| Lifestyle change | *At least one of the following*  Weight loss  Smoking cessation  Change of diet  Increase in physical activity |
| Current smoking | *At least one of the following*  Self-report  Breath carbon monoxide >10 ppm (Smokerlyzer®, Bedfont Scientific, Model Micro+) [A5] |
| Physical activity | At least 30 minutes physical activity on average 5 times a week |
| Weight status  Underweight  Normal weight  Overweight  Obese | Body mass index  <18.5 kg/m^2^  18.5-24.9 kg/m^2^  25-29.9 kg/m^2^  ≥30 kg/m^2^ |
| Cardioprotective/Anti-hypertensive medication | Beta blocker  ACE Inhibitors  Angiotensin II receptor blocker  Calcium channel blocker  Diuretics  Other anti-hypertensive drugs (alpha2-receptor agonists, alpha1-receptor blocker, direct renin-inhibitors) |
| Lipid-lowering medication | Statins  Fibrates  Cholesterol absorption inhibitors  PCSK9 inhibitors |
| Antiplatelet therapy | Aspirin  Clopidogrel  Prasugrel  Dipyridamol  Ticlopidin  Ticagrelor  Cilostazol |
| Anti-diabetic medication | Insulins  Oral sulphonylurea  Incretins  Glinides  Glitazone  SGLT2 inhibitors  Metformin  Alphaglucosidase inhibitors |

**Table S2 – Probabilities of having diabetes, stratified by study, gender, type of the index event and age**

|  | **EA-IV** | | **EA-V** | |
| --- | --- | --- | --- | --- |
|  | **Elective** | **Acute** | **Elective** | **Acute** |
| **Men** |  |  |  |  |
| **<65.0 years** | **0,14897031** | **0,24567755** | **0,04056262** | **0,09080874** |
| **65.0-69.9 years** | **0,17963829** | **0,28948224** | **0,11681946** | **0,23808631** |
| **70.0-74.9 years** | **0,27653796** | **0,41561516** | **0,04931287** | **0,10916488** |
| **≥75 years** | **0,21959981** | **0,34364334** | **0,12666206** | **0,25519395** |
| **Women** |  |  |  |  |
| **<65.0 years** | **0,07892089** | **0,13750133** | **0,02151405** | **0,04937854** |
| **65.0-69.9 years** | **0,09680848** | **0,16626953** | **0,06436232** | **0,13979424** |
| **70.0-74.9 years** | **0,15761258** | **0,25822781** | **0,02626761** | **0,05991181** |
| **≥75 years** | **0,12106323** | **0,20399662** | **0,07013614** | **0,15124109** |

**References Supplement**

A1. Petersmann A, Muller-Wieland D, Muller UA, Landgraf R, Nauck M, Freckmann G, Heinemann L, Schleicher E (2019) Definition, Classification and Diagnosis of Diabetes Mellitus. Exp Clin Endocrinol Diabetes 127(S 01):S1-S7. <https://doi.org/10.1055/a-1018-9078>

A2. Piepoli MF, Hoes AW, Agewall S, Albus C, Brotons C, Catapano AL, Cooney MT, Corra U, Cosyns B, Deaton C, Graham I, Hall MS, Hobbs FDR, Lochen ML, Lollgen H, Marques-Vidal P, Perk J, Prescott E, Redon J, Richter DJ, Sattar N, Smulders Y, Tiberi M, van der Worp HB, van Dis I, Verschuren WMM, Binno S, Group ESCSD (2016) 2016 European Guidelines on cardiovascular disease prevention in clinical practice: The Sixth Joint Task Force of the European Society of Cardiology and Other Societies on Cardiovascular Disease Prevention in Clinical Practice (constituted by representatives of 10 societies and by invited experts)Developed with the special contribution of the European Association for Cardiovascular Prevention & Rehabilitation (EACPR). Eur Heart J 37(29):2315-2381. https://doi.org/10.1093/eurheartj/ehw106

A3. Snaith RP (2003) The Hospital Anxiety And Depression Scale. Health Qual Life Outcomes 1:29. https://doi.org/10.1186/1477-7525-1-29

A4. Middleton ET, Morice AH (2000) Breath carbon monoxide as an indication of smoking habit. Chest 117(3):758-63. https://doi.org/10.1378/chest.117.3.758
